# Potential Role for PAD2 in Gene Regulation in Breast Cancer Cells

**DOI:** 10.1371/journal.pone.0041242

**Published:** 2012-07-24

**Authors:** Brian D. Cherrington, Xuesen Zhang, John L. McElwee, Eric Morency, Lynne J. Anguish, Scott A. Coonrod

**Affiliations:** 1 James A. Baker Institute for Animal Health, College of Veterinary Medicine, Cornell University, Ithaca, New York, United States of America; 2 Department of Zoology and Physiology, University of Wyoming, Laramie, Wyoming, United States of America; University Claude Bernard Lyon 1, France

## Abstract

The peptidylarginine deiminase (PAD) family of enzymes post-translationally convert positively charged arginine residues in substrate proteins to the neutral, non-standard residue citrulline. PAD family members 1, 2, 3, and 6 have previously been localized to the cell cytoplasm and, thus, their potential to regulate gene activity has not been described. We recently demonstrated that PAD2 is expressed in the canine mammary gland epithelium and that levels of histone citrullination in this tissue correlate with PAD2 expression. Given these observations, we decided to test whether PAD2 might localize to the nuclear compartment of the human mammary epithelium and regulate gene activity in these cells. Here we show, for the first time, that PAD2 is specifically expressed in human mammary gland epithelial cells and that a portion of PAD2 associates with chromatin in MCF-7 breast cancer cells. We investigated a potential nuclear function for PAD2 by microarray, qPCR, and chromatin immunoprecipitation analysis. Results show that the expression of a unique subset of genes is disregulated following depletion of PAD2 from MCF-7 cells. Further, ChIP analysis of two of the most highly up- and down-regulated genes (PTN and MAGEA12, respectively) found that PAD2 binds directly to these gene promoters and that the likely mechanism by which PAD2 regulates expression of these genes is via citrullination of arginine residues 2–8–17 on histone H3 tails. Thus, our findings define a novel role for PAD2 in gene expression in human mammary epithelial cells.

## Introduction

The post-translational conversion of positively charged arginine to neutral citrulline residues in proteins is catalyzed exclusively by the peptidylarginine deiminase (PAD) enzyme family. PAD activity is alternatively termed citrullination or deimination, and mediates wide ranging effects on protein structure, function, and protein-protein interactions 1–5.

PAD2 appears to be the ancestral PAD homologue and is widely expressed in mammalian tissues [6]. Regarding targets and function, PAD2 can citrullinate vimentin in macrophages resulting in filament network breakdown and possibly apoptosis [7]. PAD2 also citrullinates IKKγ in macrophages which suppresses NF-κB activity [8]. In the brain, PAD2 citrullinates myelin basic protein (MBP), a major component of the myelin sheath and this activity has been found to be elevated in multiple sclerosis [9], [10]. PAD2 activity has also been described in reproductive tissues. PAD2 protein is expressed in the epithelium of the uterine endometrium and the pituitary gland in rodents [11], [12]. Interestingly, PAD2 expression and citrullination activity in reproductive tissues is strongly tied to the estrous cycle, with both expression and enzymatic activity reaching their peak during the secretory phase [11]–[13].

To date, PAD4 is the only PAD family member with a documented role in gene expression. PAD4 was initially found to target histone tail arginine and methyl-arginine residues for citrullination [14]. At a promoter-specific level, PAD4 catalyzed histone citrullination has been correlated with repression of the estrogen-responsive gene *TFF1* and apoptosis-associated *p21* and *OKL38* genes [14]–[18]. In addition to gene repression, we recently carried out a genome-wide ChIP-chip study and found that PAD4 also appears to play an important role in gene transactivation in MCF-7 breast cancer cells. Further, we found that PAD4 is enriched at the Elk-1 transcription factor binding site motif within the serum response element (SRE) of the *c-fos* gene promoter [19]. This work further characterized the role of PAD4 in gene expression and defined it as a novel transcriptional co-activator.

We also recently found that PAD2 expression in the canine mammary gland initiates within a subset of epithelial alveolar cells during estrus and reaches peak expression levels during diestrus, with strong PAD2 staining detected in the nuclei at this stage. In this tissue, citrullination activity is primarily seen during diestrus and is confined to epithelial cell nuclei at this stage [20]. Interestingly, the N-termini of histone H3, but not H4, appear to be the primary target of PAD activity in canine mammary epithelium suggesting that PAD2-mediated histone citrullination may play a role in gene regulation in the mammary gland [20].

To expand upon our findings in the dog, here we illustrate for the first time that PAD2 is also expressed in human breast luminal epithelial cells. Further, we present data indicating that a portion of PAD2 localizes to the nucleus of MCF-7 cells and binds to chromatin. Using a genome-wide microarray approach, we identified a subset of genes whose expression is affected by PAD2-depletion, indicating that these genes may be targeted for regulation by PAD2. Our ChIP analysis of the most highly up- and down-regulated genes in the PAD2 knockdown line (PTN and MAGEA12) shows that PAD2 appears to bind directly to their promoters and citrullinate histone H3 arginine residues at these sites, thus further supporting the hypothesis that PAD2 regulates gene expression. Interestingly, given that both PTN and MAGEA12 are strongly associated with mammary tumor progression, our findings also suggest that PAD2 may play a role in breast cancer development. This prediction is supported by our finding that depletion of PAD2 from MCF-7 cells significantly suppresses cell proliferation.

**Figure 1:**
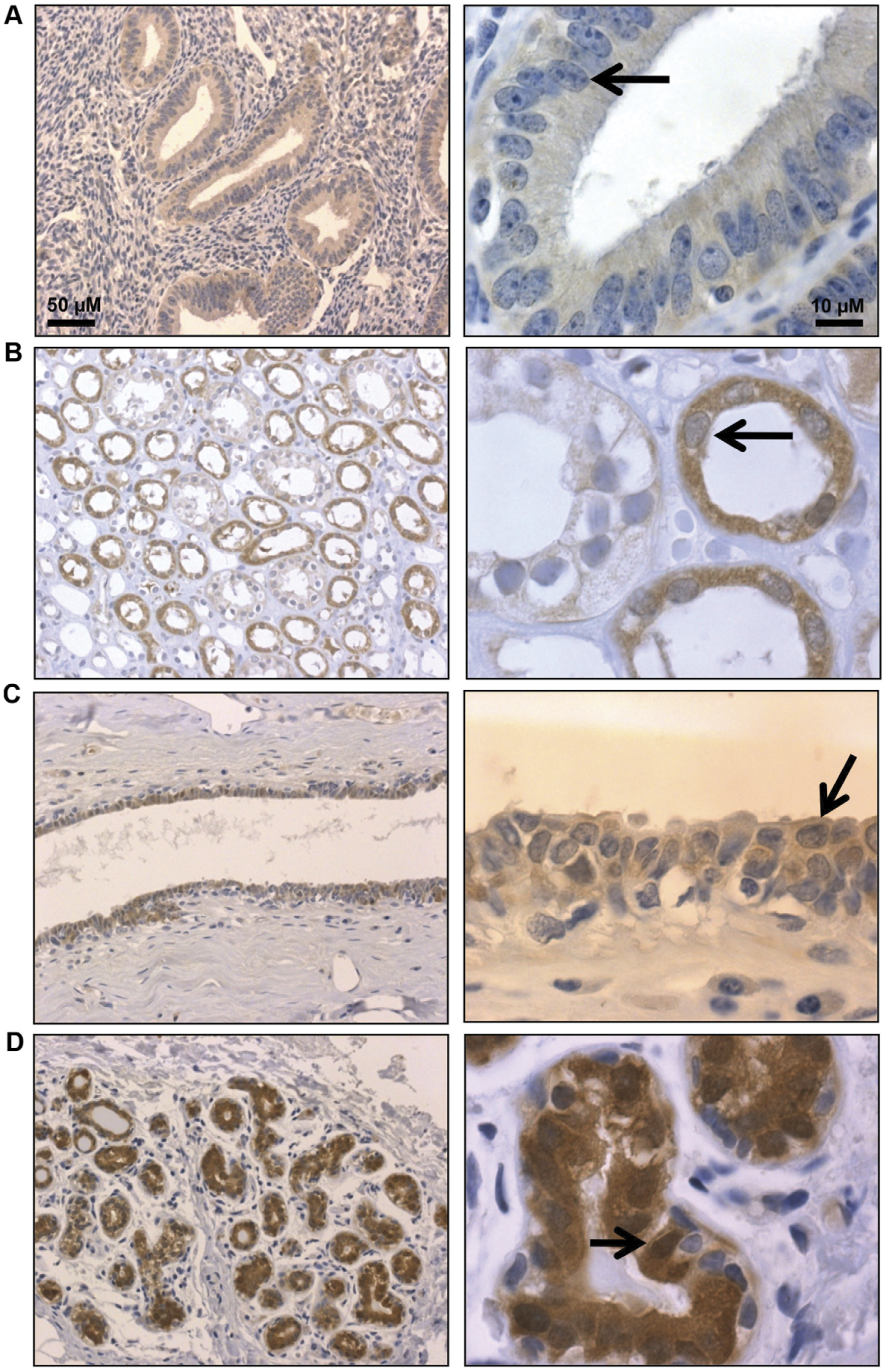
PAD2 is expressed in human endometrial, kidney, breast ductal and luminal epithelial cells. (A) Endometrial, (B) kidney, (C) mammary duct, and (D) mammary alveolar epithelial cells express PAD2, while staining is absent (or weak) in adjacent tissue. Tissue sections were probed with anti-PAD2 antibody or equal concentration of rabbit IgG as a control and counterstained with hematoxylin. The black arrows indicate epithelial cells with varying PAD2 staining in the nucleus between different tissues.

## Experimental Procedures

### Materials

Human tissue microarray slides were obtained from the Cooperative Breast Cancer Tissue Resource (National Cancer Institute) and from the Cooperative Human Tissue Network (CHTN) (University of Virginia). MCF-7 and 293 HEK cells were grown in DMEM (Invitrogen, Carlsbad, CA) supplemented with 10% fetal bovine serum (FBS) (Invitrogen), and 1% antibiotic antimycotic (Sigma-Aldrich, St. Louis, MO) and maintained in a humidified atmosphere of 5% CO_2_ at 37°C as previously described [19]. Stable shRNA control and PAD2 knockdown MCF-7 cell lines were created by transfecting cells with Sigma MISSION shRNA vectors using FuGENE6 (Roche, Pleasanton, CA). Clones were selected with 2 µg/ml of puromycin. The 437 amino acid truncated PAD2 cDNA was purchased from Open Biosystems (Hunstville, AL). The cDNA was removed from pCMV-SPORT6 by digestion with EcoRI and NotI. The resulting fragment was then ligated into pCDNA3.1+ previously digested with EcoRI and NotI and clones sequence verified. The PAD2 278 and 140 amino acid truncated isoforms were constructed following a QuikChange XL site-directed mutagenesis protocol (Agilent, Santa Clara, CA) and verified by sequencing. Primers to create 1 base pair mutations to insert stop codons were as follows: 278 5′-CTGCTGGAGTACATGGCC**T**AGGACATTCCCCTG-3′, 140 5′-CCAAAGAAGGCATCCT**A**GACCTGGGGCCCC-3′.

### Immunohistochemistry and Immunofluorescence

IHC and IF experiments were carried out using a standard protocol previously described [20]. Briefly, slides were rehydrated in 3X 5 minute washes in xylene followed by single sequential 5 minute washes in 100, 95, and 75% EtOH. Slides for IHC were then incubated for 10 minutes in 0.5% hydrogen peroxide in methanol to quench endogenous peroxidases, however this step was omitted for IF slides. Next, slides were submerged in 0.01 M sodium citrate and boiled 2X for 10 minutes to retrieve antigens. After cooling, slides were washed in 1X PBS and then blocked in 10% normal goat serum and 2X casein (Vector Labs, Burlingame, CA) for 20 minutes at room temperature in a humidified microprobe chamber. Next, slides were blotted to remove excess blocking solution and then primary antibody diluted in 1X PBS was added for 2 hours at room temperature. Antibody dilutions are as follows: anti-PAD2 1∶100 (ProteinTech #122100-1-AP, Chicago, IL), anti-Cytokeratin 1∶100 (Dako MS3515, Carpinteria, CA), anti-H3K9 acetyl 1∶200 (Abcam, ab12179, Cambridge, MA), anti-HP1α 1∶200 (Millipore, #05-689, Billerica, MA), anti-H3K9 dimethyl 1∶200 (Abcam, ab1220), anti-H3K27 trimethyl 1∶100 (Abcam, ab6002), anti-PAD4 1∶100 (Sigma, P4749), anti-FLAG M2 1∶200 (Sigma, F1804). After washing three times, slides were incubated with a secondary antibody (biotinylated secondary for PAD2) diluted 1∶200 in 1X PBS for 1 hour at room temperature then washed 3X in PBS. IHC slides were incubated in DAB chromagen (Vector Labs) solutions according to the manufacturer’s protocol, washed and then counterstained with hematoxylin and coverslip mounted. IF slides were incubated in streptavidin conjugated-488 (Invitrogen), washed and then mounted using Vectashield containing DAPI (Vector Labs). During each experiment, duplicate slides were treated with control rabbit or mouse IgG antibody at the appropriate concentrations as a negative control.

**Figure 2:**
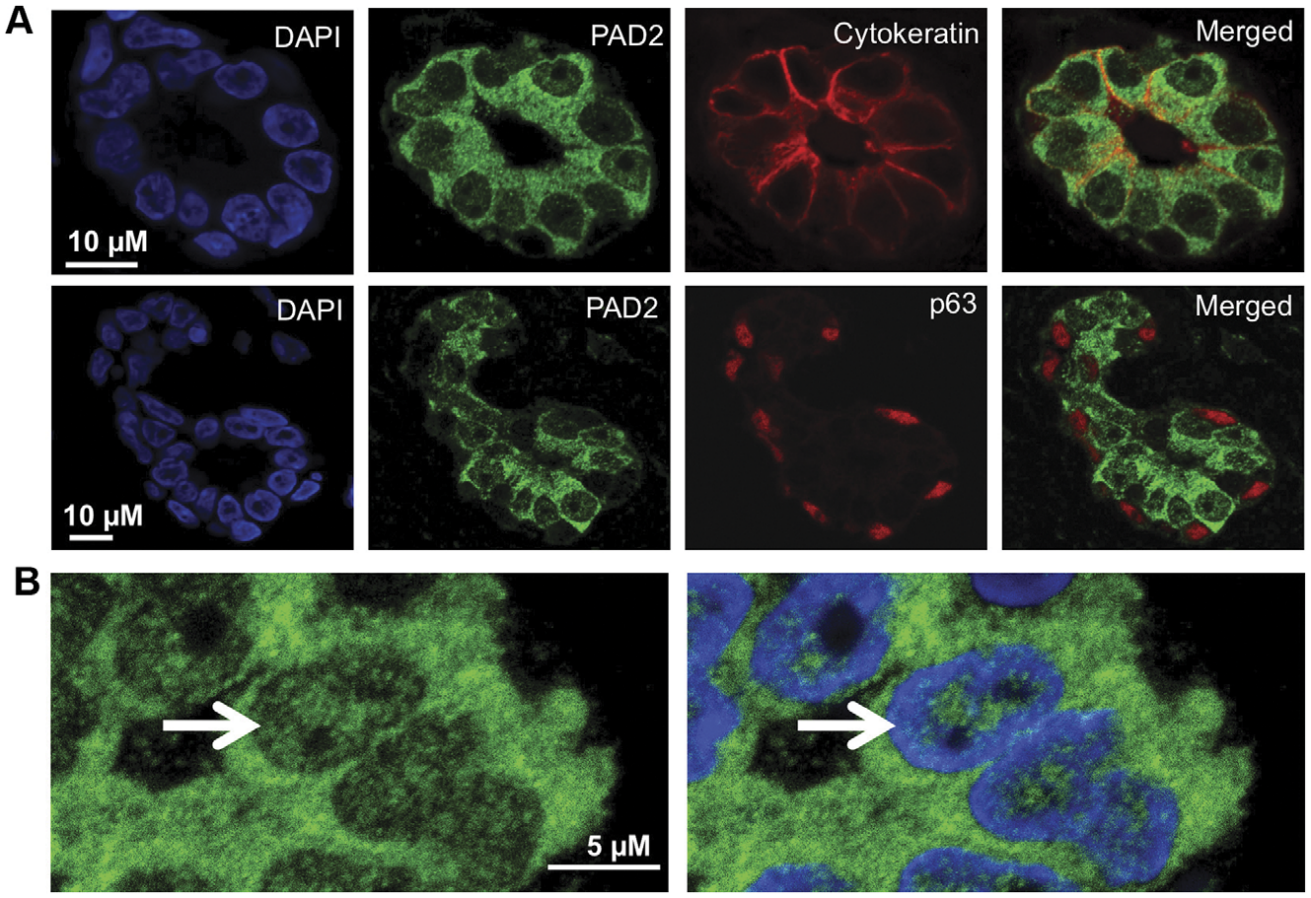
PAD2 is detected in the cytoplasm and nucleus of human breast luminal epithelial cells. (A) PAD2 is expressed in luminal but not myoepithelial human breast cells. Normal human breast tissue sections were probed with anti-PAD2 (green fluorescent signal), anti-cytokeratin (luminal marker - red fluorescent signal), and anti-p63 (myoepithelial marker - red fluorescent signal) antibodies or equal concentration of rabbit and mouse IgG as a control and nuclei are stained with DAPI. (B) A portion of PAD2 staining in human breast epithelial cells is detected in the nucleus. Normal human breast tissue sections were probed with anti-PAD2 (green fluorescent signal) and nuclei are stained with DAPI. The white arrow illustrates a breast epithelial cell with robust PAD2 staining in the nucleus.

### Western Blotting, Chromatin Fractionation, and Cell Proliferation Assays

Cells were lysed in RIPA buffer containing 20 mM Tris (pH 8.0), 137 mM NaCl, 10% glycerol, 1% NP-40, 0.1% SDS, 0.5% deoxycholate, and 0.2 mM PMSF and 1X general protease inhibitor. Protein concentration in lysates was determined by Bradford Assay prior to gel loading to ensure equal protein loading. 6X sample buffer (300 mM Tris-HCl, pH 6.8, 60% glycerol, 30 mM DTT, 6% SDS) was added to yield a final concentration of 1X and lysates were boiled at 95°C for 5 min. Samples were subjected to SDS polyacrylamide gel electrophoresis on a 10% gel (acrylamide:bis-acrylamide ratio of 29∶1) and electro-blotted to Immobilin PVDF membranes (Millipore). Membranes were blocked in 1X casein diluted in Tris buffered saline (TBS). Anti-PAD2 1∶1000 (ProteinTech) and anti-FLAG M2 1∶4000 (Sigma), were incubated overnight at 4°C. Blots were washed and then incubated with a 1∶10,000 dilution of anti-rabbit or mouse conjugated HRP (Jackson ImmunoResearch Labs, West Grove, PA) for 2 hr at room temperature. All blots were washed for 60 min (6×10 min) with TBS-Tween after secondary antibody and then visualized by chemiluminescence using Millipore Immobilon Western. To confirm equal protein loading, membranes were stripped and re-probed with anti-β-actin (Abcam, ab8227). Chromatin fractionation was carried out as previously described [21]. Protein concentrations for resulting fractions were determined by Bradford Assay and subject to western blot analysis as described above. Cleanliness of fractionation was determined by stripping membranes and re-probing with anti-TBP 1∶1000 (Abcam, ab818) or anti-Histone H3 1∶1000 (Millipore, 06–755) and SOD4 1∶1000 (Abcam, ab16962). Cell proliferation studies on control versus PAD2 depleted MCF-7 cells were carried out using the standardized non-radioactive cell proliferation assay protocol (MTT) from Promega (Madison, WI). Briefly, ∼5000 cells/well were plated in triplicate and allowed to grow for 24, 48 or 72 hours. Following incubation with the dye solution, formazan product was measured at 570 nm.

**Figure 3:**
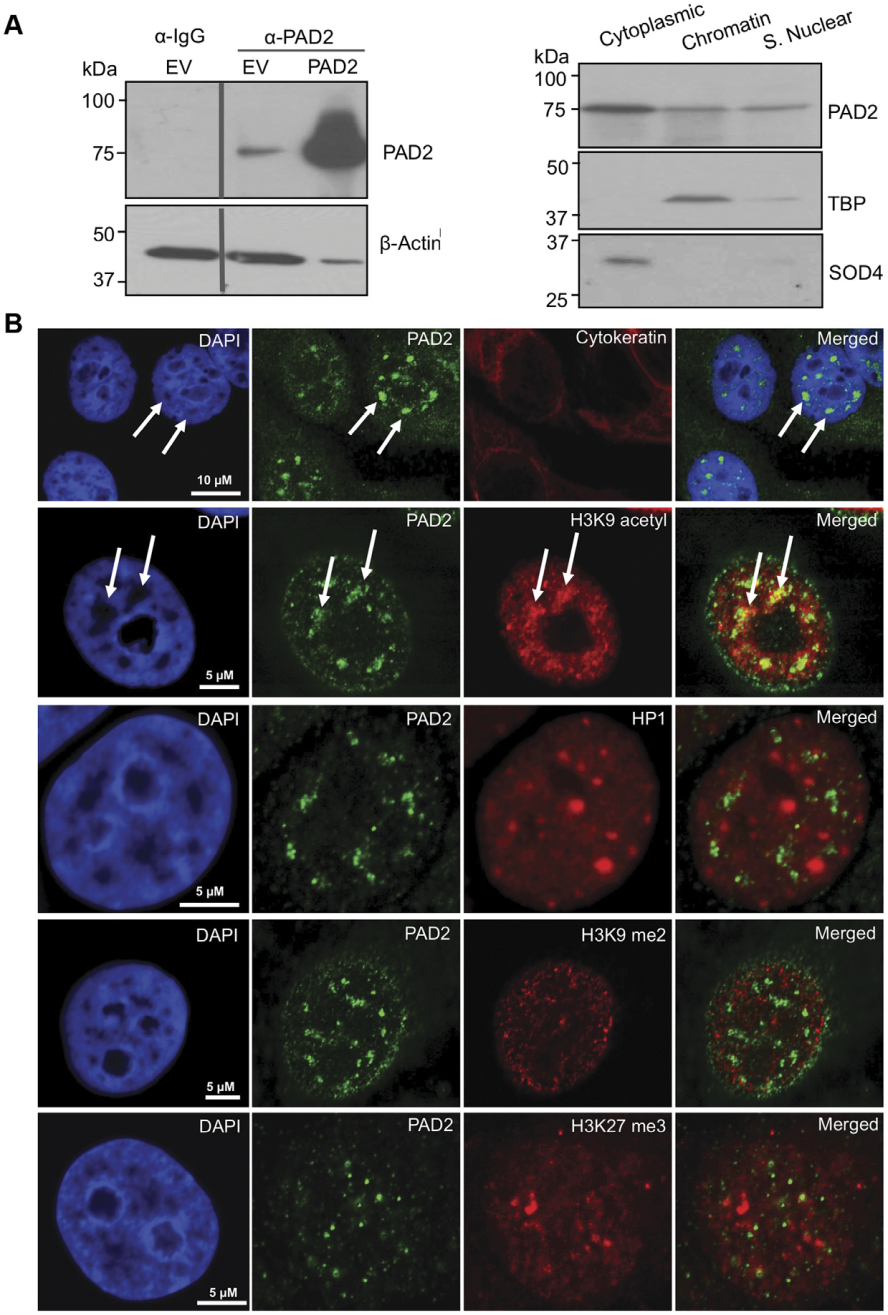
PAD2 associates with chromatin in the nucleus of MCF-7 cells. (A) PAD2 is endogenously expressed in MCF-7 cells and is detected in multiple cellular compartments. Wild type and PAD2 over-expressing MCF-7 whole cell lysates were subject to SDS-PAGE and probed with an anti-PAD2 antibody. Anti-Rabbit IgG was used as a negative control. (left panel). Endogenous MCF-7 cellular proteins were also separated into cytoplasmic, chromatin, and soluble nuclear pools by fractionation methods and examined by western blot (right panel). Cleanliness of fractionation was determined by stripping membranes and re-probing with antibodies for TBP (nuclear) and SOD4 (cytoplasmic) proteins. (B) Punctate PAD2 expression is detected in the nucleus of MCF-7 cells. MCF-7 cells were subject to IF using anti-PAD2 (green), anti-cytokeratin (luminal-red), anti-H3K9 acetyl (euchromatin-red), anti-HP1 (heterochromatin-red), anti-H3K9 dimethyl (facultative heterochromatin-red), and anti-H3K27 trimethyl (facultative heterochromatin-red) antibodies while DAPI nuclear stain is in blue. White arrows denote punctuate PAD2 staining in DAPI poor nuclear regions.

### qPCR

RNA was purified from MCF-7 cells according to the Qiagen RNeasy protocol including on-column DNAse treatment to remove genomic DNA. The resulting RNA was quantified and 0.5 µgs reverse transcribed using ABI High Capacity RNA to cDNA kit according to manufacturer’s protocol (ABI, 4387406, Foster City, CA). Complementary DNA was subject to qPCR analysis with TaqMan assays from ABI for PAD2 (Hs00247108_m1), PAD4 (Hs00202612_m1), and GAPDH (4352934E). Data was analyzed by the delta/delta Ct method in which all target gene Ct values are adjusted to corresponding reference gene Ct levels. Stable MCF-7 shRNA scrambled control Ct values were normalized to 1 and all values are expressed as the mean ± SEM. Means were separated using Student’s T-Test with * indicating means significantly different (P<0.05) from shRNA control. For microarray qPCR validation experiments, cDNA was generated as described above and subject to qPCR using the primers listed in Table S1. Data was analyzed using the ABI Data Assist Software v3.0 and P value set to P<0.05.

### Gene Expression Microarray

Total RNA from shRNA control and PAD2 depleted MCF-7 cells was prepared using RNeasy mini kit (Qiagen) from four independent biological replicates. Microarray studies and data analysis was performed by the Cornell University Life Sciences Core Laboratories Center. Briefly, RNA was assessed for quality using an Agilent Bioanalyzer 2100. Labeling was carried out using the Amino Allyl MessageAmpTM II ARNA amplication kit from Ambion following the manufacture’s protocol. Hybridization followed a one-color microarray-based gene expression analysis, low input quick amp labeling protocol from Agilent (Santa Clara, CA). Slides were scanned immediately after washing using an Agilent DNA Microarray Scanner (G2505B), and the scanned images were analyzed with Feature Extraction Software 9.1 (Agilent). Data was analyzed with Ingenuity Systems IPA version 8.8 and significant genes determined using P<0.001 with a q value (FDR) of <0.01. Microarray experiments were compiled using MIAME guidelines and the resulting dataset has been deposited into the NCBI GEO database (GSE37087).

### Chromatin Immunoprecipitation

Chromatin immunoprecipitation (ChIP) was performed essentially as described previously [22]. Briefly, stable FLAG-tagged PAD2 overexpressing, shRNA control, and PAD2 depleted MCF-7 cells were grown to ∼80 to 90% confluence, cross-linked with 1% paraformaldehyde for 10 min at 37°C, and quenched in 125 mM glycine for 5 min at 4°C. The cells were lysed (1% SDS, 10 mM EDTA, 50 mM Tris·HCl, pH 7.9, 1x protease inhibitor cocktail) and sonicated under conditions yielding fragments ranging from 200bp to 800bp. The material was clarified by centrifugation, diluted 10-fold in dilution buffer (0.5% Triton X-100, 2 mM EDTA, 150 mM NaCl, 20 mM Tris·HCl, pH 7.9, 1x protease inhibitor cocktail), and pre-cleared with protein A-agarose beads. The pre-cleared, chromatin-containing supernatant was used in immunoprecipitation reactions with FLAG-M2 beads (Sigma Aldrich) or protein A-agarose beads loaded with anti-H4 cit 3 (Milipore, 07–596) or anti-H3 cit 2–8–17 (Abcam, ab77164). Ten percent of the supernatant was saved as reference control. The immunoprecipitated genomic DNA was cleared of protein and residual RNA by digestion with proteinase K and RNase (Roche), respectively. The DNA was then extracted with phenol:chloroform:isoamyl alcohol and precipitated with ethanol. For gene-specific ChIP analyses, quantitative real-time PCR (qPCR) was used to determine the enrichment of immunoprecipitated DNA relative to the input DNA using gene-specific primer sets (Table S1). Each ChIP experiment was conducted a minimum of three times with independent chromatin isolates to ensure reproducibility.

**Figure 4:**
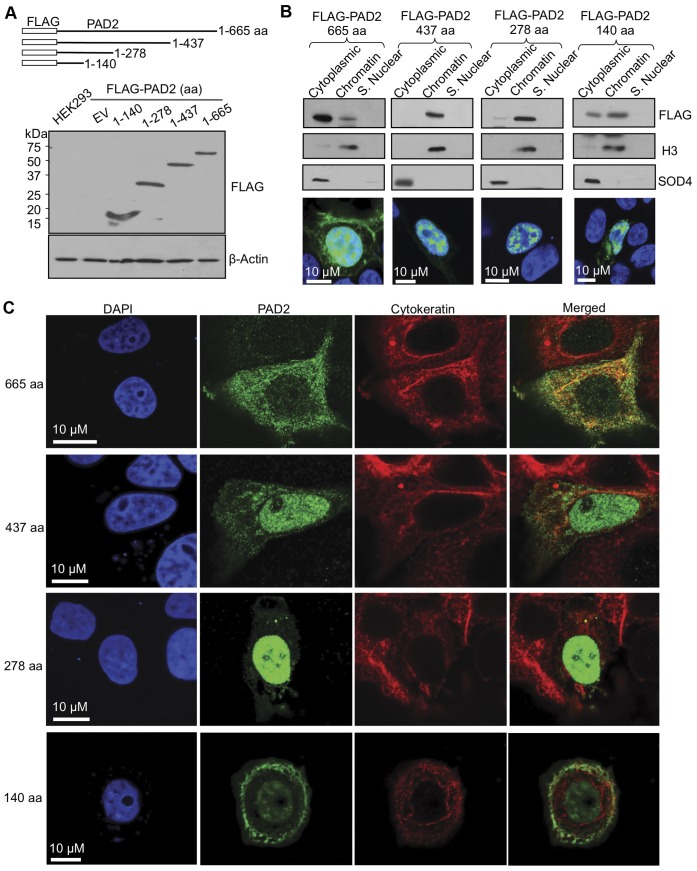
Truncated FLAG-tagged PAD2 proteins reveal regions necessary for nuclear localization. (A) The schematic at the top indicates the different truncated PAD2 proteins of 140, 278 and 437 amino acids. Truncated FLAG-PAD2 constructs were validated by overexpressing in HEK 293 cells with lysates analyzed by SDS-PAGE. Membranes were probed with an anti-FLAG antibody which detected predicted molecular weight of truncated proteins while β-actin shows loading control. (B) Truncation of PAD2 reveals regions of the protein necessary for subcellular localization by cellular fractionation. Truncated FLAG-PAD2 constructs were overexpressed in HEK 293 cells after which cellular proteins were separated by fractionation methods and examined by western blot. Cleanliness of fractionation was determined by stripping membranes and re-probing with antibodies for Histone H3 (nuclear) and SOD4 (cytoplasmic) proteins. Truncated FLAG-PAD2 constructs were also transiently transfected into HEK 293 cells and subcellular localization examined by IF (bottom panel of B) using an anti-FLAG antibody (green fluorescent signal) to corroborate cellular fractionation studies. (C) Truncation of PAD2 reveals regions of the protein necessary for subcellular localization by IF in mammary epithelial cells. Truncated FLAG-PAD2 constructs were over expressed in MCF-7 cells and examined by IF using an anti-PAD2 (green) and anti-cytokeratin (red) antibodies while DAPI nuclear stain is in blue.

### Statistical Analysis

All experiments were independently repeated at least three times. Values were expressed as the mean ± the SEM. Means were separated using Two-tailed Student’s T-Test with * indicating means significantly different (P<0.05) from control and ** indicating P<0.01.

**Figure 5:**
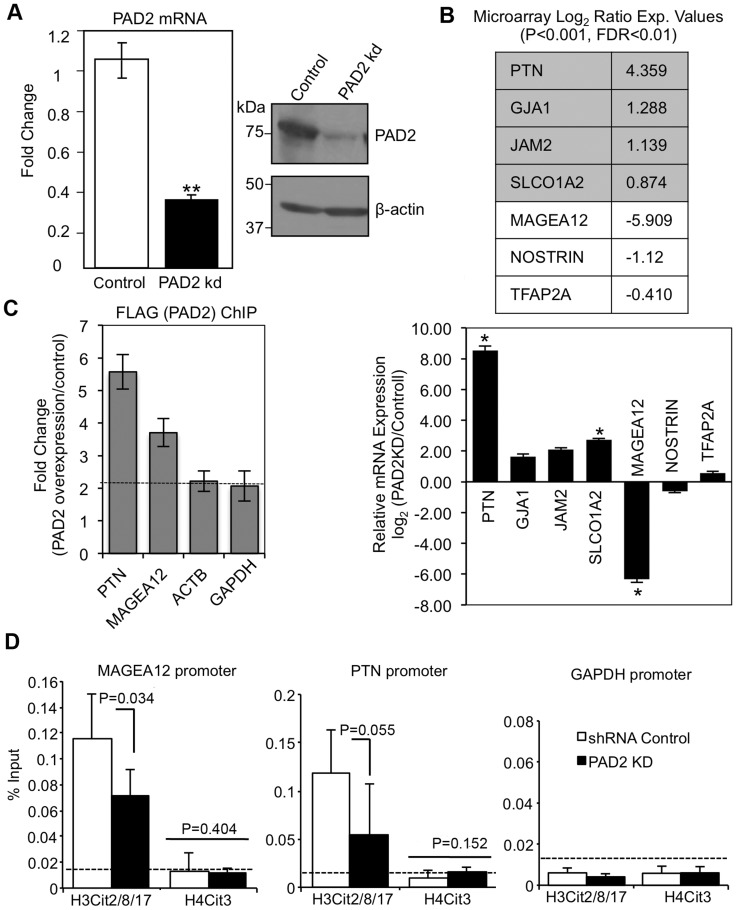
PAD2 plays a role in gene expression in MCF-7 cells. (A) PAD2 levels are significantly reduced in PAD2 knock down MCF-7 cells compared to shRNA controls. Whole cell lysates from shRNA control and PAD2 knock down MCF-7 cells were analyzed by western blot using an anti-PAD2 antibody and an anti-β-actin antibody for loading control. RNA was purified from PAD2 knock down MCF-7 and shRNA control cells, reverse transcribed, and resulting cDNA used in qPCR reactions containing TaqMan assays to PAD2 and GAPDH as the reference gene control. Data represent the means ± SEM of four independent experiments performed in triplicate. All values are normalized to shRNA control samples and bars represent the means ± SEM. Means were separated by Student’s T-Test and ** represent significant differences (P<0.01). (B) Microarray and qPCR validation studies indicate that PAD2 is involved in gene regulation in MCF-7 cells. The table shows a sampling of genes whose expression profiles were significantly altered between shRNA control and PAD2 knockdown MCF-7 cells. Numbers represent log_2_ ratio of fold change of expression between control and PAD2 knockdown cell lines with P<0.001 and FDR<0.01. To validate microarray studies, total RNA was harvested from shRNA control and PAD2 knockdown MCF-7 cells and reverse transcribed. Resulting cDNA was used in qPCR reactions with specific primers listed in Table S1. The graph shows fold change of log_2_ ratio of expression between control and PAD2 knockdown cell lines with P<0.05. (C) PAD2 associates with the PTN and MAGEA12 gene promoters in MCF-7 cells stably overexpressing FLAG-tagged PAD2. ChIP-qPCR studies were done using an anti-FLAG antibody and data is presented as fold change of signal from MCF-7 cells overexpressing FLAG-tagged PAD2 over control cells with β-actin and GAPDH serving as controls. Data represent the means ± SEM of three independent experiments. (D) The PTN and MAGEA12 gene promoters show citrullination of histone H3, but not histone H4 tail arginine residues using shRNA control and PAD2 knockdown MCF-7 cells. ChIP-qPCR studies were done using an anti-H3 cit 2–8–17 and H4 cit 3 antibodies and data is presented as % input with the GAPDH promoter serving as the control. Data represent the means ± SEM of three independent experiments and means separated by one-tailed T-tests.

## Results

### Human PAD2 is Expressed in the Luminal Epithelium of Mammary Lobulo-alveolar Units

Currently, relatively little is known regarding PAD2 expression patterns in normal human tissues. Therefore, we first probed a human tissue array by immunohistochemistry (IHC) using a previously validated anti-PAD2 antibody (20) or an equal mass of rabbit IgG as a control (Figure S1) and determined that PAD2 is primarily expressed in the cytoplasm of epithelial cell populations in the human endometrium, kidney, mammary duct, and mammary lobulo-alveolar units (Figure 1A, B, C, D). Of these tissues, we found that the strongest PAD2 signal was observed in the mammary gland and was largely confined to the epithelium of the terminal ductal-lobular unit (Figure 1D). Interestingly, higher magnification revealed that a significant fraction of PAD2 also appears to localize to the nucleus in the mammary epithelium (Figure 1D arrows).

Our immunofluorescence (IF) studies in the dog found that PAD2 was predominately expressed in luminal (cytokeratin), but not myoepithelial (p63) mammary epithelial cells in this species. We conducted similar experiments on human breast tissue and found that PAD2 retained the luminal localization pattern in humans (Figure 2A). Further, at higher magnification the localization of PAD2 to the nucleus of human mammary epithelial cells was evident. We also found that PAD2 mainly appears to localize to regions of the nucleus that stain poorly for DAPI suggesting that PAD2 may be primarily associated with euchromatin (Figure 2B).

**Figure 6:**
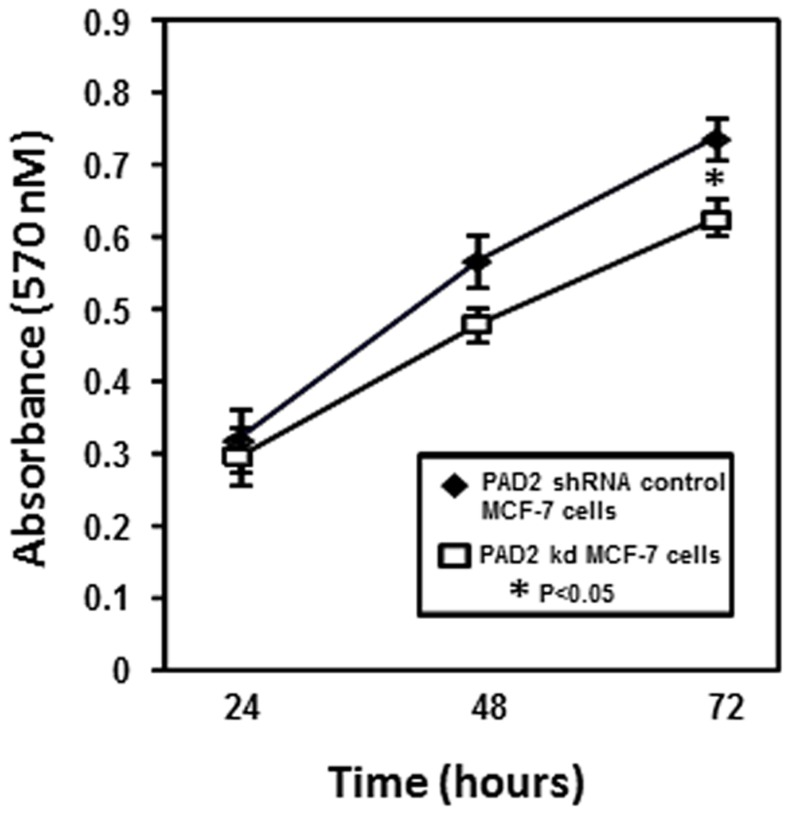
Knockdown of endogenous PAD2 in MCF-7 cells alters cellular proliferation rates versus control cells. Equal numbers of shRNA control and PAD2 knockdown MCF-7 cells were plated in 96-well plates and allowed to grow for 24, 48, or 72 hours. At time of harvest, dye solution from MTT assay kit was allowed to incubate with cells for one hour at which point solubilization reagent was added and resulting formazan product measured at 570 nm. Data represent the means ± SEM of three independent experiments performed in triplicate. Values represent the means ± SEM.

### Endogenous PAD2 Fractionates, in Part, with Chromatin in the MCF-7 Breast Cancer Cell Line

To further test the hypothesis that PAD2 localizes to the nucleus and possibly plays a role in gene regulation, we next continued our studies using the luminal subtype MCF-7 breast cancer cell line. We first demonstrated by western blotting that PAD2 is expressed in this cell line using an anti-PAD2 antibody that is not cross-reactive with other PAD family members (Figure 3A, left panel, Figure S3A) (20). Next, we resolved endogenous PAD2’s subcellular localization within MCF-7 cells by small-scale chromatin fractionation, which separates cellular proteins into three distinct pools: cytoplasmic, chromatin, and soluble nuclear proteins. We found that PAD2 is detected in all three subcellular compartments in MCF-7 cells, with a portion of PAD2 being associated with the chromatin fraction (Figure 3A, right panel, Figure S3). Accuracy of cell fractionation was confirmed by stripping the membranes and re-probing for Superoxide Dismutase 4 (SOD4), a cytoplasmic protein, and TATA Binding Protein (TBP), a nuclear protein. To further refine PAD2’s nuclear localization in MCF-7 cells, we used antibodies to histone modifiers and modifications, which have been previously shown to localize to specific sub-nuclear domains. As with primary mammary epithelial tissue, we found that PAD2 localizes to DAPI-poor regions of the nucleus (Figure 3B 1). While little co-localization was observed between PAD2 and the repressive histone markers (H3K9Me2, HP1, and H3K27Me3), partial co-localization was found between PAD2 and the euchromatic marker, H3K9 acetyl, thus supporting the hypothesis that PAD2 associates with euchromatin in the nucleus (Figure 3B 2–5).

### Truncated Mutants Reveal the N-terminus of PAD2 is Important for Targeting to the Nucleus

Next, we examined which domains within PAD2 are important for targeting PAD2 to the nucleus using the highly transfectable Human Embyonic Kidney (HEK) 293 cell line. In addition to examining a full length FLAG-tagged PAD2 expression vector (665 aa), premature stop codons were inserted into a FLAG-tagged 437 amino acid PAD2 isoform to create truncated PAD2 proteins of 278 and 140 amino acids and these constructs were then transiently transfected into HEK 293 cells. Following sequencing and confirmation that the mass of these PAD2 proteins corresponded to their predicted molecular weight (Figure 4A, Figure S4), the constructs were then transfected into HEK 293 cells and subjected to chromatin fractionation. Supporting our endogenous PAD2 fractionation studies in MCF-7 cells, full length FLAG-tagged PAD2 is detected in the cytoplasmic and the chromatin fraction of 293 cells. Interestingly, truncation of PAD2 to 437 or 278 amino acids results in localization only in the chromatin fraction, suggesting that a motif between 665 and 437 amino acids is important for retention of PAD2 in the cytoplasm, and thus prevents PAD2 from being exclusively targeted to chromatin (Figure 4B, Figure S4). Further, a region located in the first 437 amino acids is sufficient for nuclear localization; however truncation to 140 amino acids results in a reacquisition of PAD2 in the cytoplasm. Chromatin fractionation studies were further validated by transiently transfecting HEK 293 cells with the FLAG-tagged PAD2 truncation mutants and examining them by IF which corroborated fractionation studies (Figure 4B bottom panel). Since differences in subcellular localization and staining intensity could be seen between kidney (Figure 1B) and normal breast tissue (Figure 1D) in IHC, we also wanted to examine PAD2’s subcellular localization in mammary epithelial cells and, thus, examined transiently transfected MCF-7 cells by IF. Results show that as PAD2 is truncated from full length to 1–437 and then to 1–278 amino acids, there is a progressive concentration of PAD2 signal within the nuclear compartment (Figure 4C). Although the PAD2 truncation of 140 amino acids retains nuclear localization, an increasing amount of PAD2 is again detected in the cytoplasm of cells. These results further support the hypothesis that PAD2 is associated with chromatin within the cell nucleus.

### PAD2 Regulates Gene Expression and Likely Promotes Cell Proliferation

Since PAD2 associates with chromatin, we next asked if PAD2 plays a role in mediating gene expression. To address this question, we created stable shRNA control and PAD2 depleted MFC-7 cell lines. Validation of the cell lines revealed that PAD2 mRNA and protein levels were significantly reduced in the knock down cell line compared to the shRNA scrambled control (Figure 5A, Figure S5). Due to the high degree of homology between PAD2 and PAD4, we also examined PAD4 mRNA and proteins levels and found that PAD4 levels were not significantly reduced (Figure S2). Next, we examined genome-wide differences in the gene expression patterns between the control and PAD2 knockdown MCF-7 cell lines. Microarray data identified a number of candidate genes that were either up- or down-regulated in the control compared to PAD2 knockdown MCF-7 cells with P<0.001and a false discovery rate of <0.01 (Figure 5B). We also examined our microarray data to examine the levels of TFF1 and p21, known targets of PAD4 mediated gene regulation. Neither PAD4 target gene was differentially regulated in the PAD2-depleted versus control MCF-7 cell line raising the possibility that PAD2 may target different gene promoters compared to PAD4. Of the candidate genes identified in our microarray screen, we picked a range of significantly up and down regulated genes to validate by qPCR (Table S1). QPCR validation studies corroborated microarray results showing that expression of PTN, GJA1, and SLCO1A2 are significantly upregulated while MAGEA12 is significantly down regulated (P<0.05) (Figure 5B). These studies are the first to suggest that PAD2 may play a role in gene regulation in MCF-7 cells.

We next used chromatin immunoprecipitation (ChIP) analysis to determine if PAD2 can associate with gene promoters in cells. For initial ChIP assays we tried multiple anti-PAD2 antibodies using the shRNA control and PAD2 knock down MFC-7 cell lines, however none of the antibodies showed sufficient ability to immunoprecipitate chromatin. Thus, we created a stable MCF-7 cell line that overexpresses FLAG-tagged PAD2 and used this cell line for ChIP analysis. We chose to examine if PAD2 can associate with the PTN and MAGEA12 gene promoters as these two genes showed the largest differential regulation in control versus PAD2 depleted cells in our microarray and qPCR validation studies (Figure 5B). Our results show an increase in PAD2 enrichment on both the PTN and MAGEA12 gene promoters in the FLAG-PAD2 MCF-7 cells compared to the control cell line (Figure 5C, Table S1). Given our previous observation that histone citrullination correlated with PAD2 expression across the estrous cycle in the canine mammary epithelium (20), we next tested whether PAD2 might regulate gene activity via histone tail citrullination. To do this we compared citrullination levels on histone H3 and H4 tails at the PTN and MAGEA12 gene promoters in both wild type and PAD2-depleted MCF-7 cells. Results show that depletion of PAD2 results in a decrease in citrullination on the histone H3 tail (at arginine residues 2–8–17) on both the MAGEA12 and PTN gene promoters while citrullination at arginine 3 on histone H4 was not affected (Figure 5D). Interestingly, we also found that the growth rate of the PAD2-depleted MCF-7 cell line was significantly reduced (Figure 6), thus raising the possibility that the observed PAD2-mediated gene regulatory activity may facilitate cell proliferation.

## Discussion

Our previous work identified a novel role for PAD2 in the canine mammary gland by showing that PAD2 likely targets epithelial cell histones for citrullination during the diestrus phase of the estrous cycle. Based on this novel finding, we expanded our studies here to test whether PAD2 might associate with chromatin and regulate gene activity in human breast epithelial cells. Strikingly, we found that PAD2 is robustly expressed in the luminal epithelium of terminal ductal-lobular units in human mammary tissue and localizes, in part, to the nucleus of these cells. Using tissue culture, we then confirmed PAD2’s nuclear localization and also demonstrated that a portion of endogenous PAD2 partitions in the chromatin fraction of MCF-7 cells. Given this observation, we next attempted to refine the localization of PAD2 to distinct sub-populations of chromatin. We found that PAD2 signal was detected in DAPI poor regions of the MCF-7 cell nucleus, suggesting that PAD2 primarily localizes to euchromatin within the nucleus. This prediction is supported by our finding that PAD2 does not co-localize with markers for heterochromatin (HP1), or facultative heterochromatin (H3K9 me2 and H3K27 me3) but does partially co-localize with the euchromatic marker, H3K9acetyl. Investigations are currently underway to further refine the localization of PAD2 within the nucleus.

To expand upon these findings, we examined a full length 665 amino acid FLAG-tagged PAD2 protein expression vector and truncated FLAG-tagged PAD2 isoforms to identify regions of the protein that are important for targeting to chromatin. Given that the PAD4 crystal structure has been resolved and the high sequence homology between PAD4 and PAD2, we placed stop codons into the PAD2 sequence based on defined regions in PAD4 [23]. Truncation of PAD2 from 665 to 437 and to 278 amino acids resulted in increasing proportions of PAD2 being targeted to the cell nucleus. This finding suggests that a region of PAD2 from 438–665 may be important for cytoplasmic retention, potentially via protein-protein interaction, while a motif in the first 437 amino acids is sufficient for nuclear localization. Interestingly, truncation from 437 to 278 amino acids increases nuclear localization, while truncation to 140 amino acids retains nuclear localization but reacquires cytoplasmic expression, indicating that more than one motif may be necessary for subcellular localization.

Our studies here utilized IIF, IHC, and most importantly, small scale chromatin fractionation to document that both endogenous and FLAG-Tagged PAD2 appear to localize to the nucleus and bind to chromatin. These well-accepted techniques, however, are not without limitation and future studies will likely need to be carried out to unequivocally confirm the presence of PAD2 in the nucleus. While much of the previous work on PAD2 has been biochemical in nature, one previous study by Nakashima et al. overexpressed GFP-tagged forms of PADs 1, 2, 3, and 4 in HeLa cells and concluded that PAD4 was the only PAD member that localized to the cell nucleus, likely via its canonical nuclear localization signal (NSL) [24]. It is not clear why our results differ from those of Nakashima, although the GFP tag, which is considerably larger than that of the FLAG tag, might have hindered the ability of the PAD2 fusion protein to be targeted to the nucleus. In further support of our conclusion that a portion of PAD2 localizes to the nucleus, a recent report by Jang *et al.* showed by cell fractionation and immunogold electron microscopy that PAD2 is found in both the cytoplasmic and nuclear compartment of mouse astrocytes and neurons [25]. Given that PAD2 does not possess a canonical NLS, it is currently unclear how PAD2 enters and is retained in the nucleus. While speculative, we hypothesize that either a distinct nuclear directing motif in the PAD2 coding sequence or an interaction between PAD2 and an NLS-containing protein may function to target and/or retain PAD2 in the nucleus. In support of this hypothesis, signaling proteins such as ERKs, MEKS, and SMADS translocate to the nucleus upon stimulation via an NLS-independent mechanism [26], [27] while protein-protein interactions like the one between tissue transglutaminase (tTG) and vascular endothelial growth factor receptor 2 (VEGFR-2) can also facilitate nuclear import upon stimulation [28]. A recent study has shown the functional relevance of PAD4 dimerization [29]. Given the high degree of homology between PAD2 and PAD4 isoforms, it is intriguing to speculate that heterodimerization may occur between PAD2 and PAD4; thus, PAD2 may enter the nucleus by dimerizing with PAD4. Studies are currently underway to test this hypothesis.

Given our finding that PAD2 associates with chromatin in MCF-7 cells, we next focused our attention on investigating the role of PAD2 in this subcellular compartment. To do this, we first used a non-biased, genome-wide approach to compare the gene expression profiles of wild type and PAD2-depleted MCF-7 cell lines and then validated candidate genes by qPCR. We found the most highly up-regulated gene in PAD2-depleted cells to be pleotropin (PTN), a secreted heparin-binding growth factor believed to be important in tumor angiogenesis and also described as a proto-oncogene [30], [31]. We found the most highly down-regulated gene in the PAD2-depleted cells to be melanoma associated antigen A12 (MAGEA12), a member of the large MAGE family of proteins that have been observed in brain, liver, lung, prostate, ovarian, skin and thyroid cancers [32], [33]. Interestingly, these proteins are normally only expressed in testis germline cells and aberrant expression of these factors is believed to promote survival, proliferation, and metastasis of cancer cells [34], [35]. To address if PAD2 directly regulates the expression of the PTN and MAGEA12 genes, we performed ChIP assays using MCF-7 cells stably overexpressing a FLAG-tagged version of full-length PAD2. This approach was necessary as ChIP-quality antibodies to PAD2 are currently not available. Results from these ChIP experiments found that PAD2 binds both the PTN and MAGEA12 promoters, thus supporting a role for PAD2 in gene regulation *in vivo*. Given that PAD4 has been found to regulate gene activity by citrullinating specific arginine residues on histone H3 and H4 tails, we next tested whether PAD2 might also regulate the expression of MAGEA12 and PTN via similar mechanisms. Our ChIP studies using previously validated site-specific anti-citrullinated histone antibodies found that citrullination was suppressed at histone H3 arginine residues (2–8–17) on the MAGEA12 and PTN gene promoters in PAD2-depleted cells while citrullination at histone H4 arginine 3 was not observed in either cell line. This finding supports our previous observations in canine mammary tissue (20) and defines a new role for PAD2 as an epigenetic modifier of gene activity via histone tail citrullination.

Regarding a potential function of PAD2-mediated gene regulation in mammary epithelial cells, we found that PAD2 depletion suppresses MCF-7 cell proliferation rates when compared to the shRNA control cells (Figure 6). Interestingly, the two genes found in this study to be most strongly affected by PAD2 depletion, MAGEA12 and PTN, are closely associated with tumor progression in a range of cancers (including breast cancer) (30–35). Therefore, our findings raise the possibility that PAD2-mediated citrullination of the histone H3 tail at MAGEA12, PTN, and likely other promoters, plays a role in breast cancer cell proliferation.

## Supporting Information

Figure S1 **PAD2 staining in human mammary tissue is specific.** As a control, human mammary tissue sections were probed with rabbit IgG at a concentration equal to that of primary PAD2 antibody and counterstained with hematoxylin.
(TIF)

Figure S2 **PAD4 expression levels are not altered in PAD2 knock down MCF-7 cells.** Whole cell lysates from shRNA control and PAD2 knock down MCF-7 cells were analyzed by western blot using an anti-PAD4 antibody and an anti-β-actin antibody for loading control. RNA was purified from PAD2 knock down MCF-7 and shRNA control cells, reverse transcribed, and resulting cDNA used in qPCR reactions containing TaqMan assays to PAD4 and GAPDH as the reference gene control. Data represent the means ± SEM of four independent experiments performed in triplicate. All values are normalized to shRNA control samples and bars represent the means ± SEM. Means were separated by Student’s T-Test (P<0.01).
(TIF)

Figure S3 **PAD2 associates with chromatin in the nucleus of MCF-7 cells.** (A) PAD2 is endogenously expressed in MCF-7 cells and is detected in multiple cellular compartments. Wild type and PAD2 over-expressing MCF-7 whole cell lysates were subject to SDS-PAGE and probed with an anti-PAD2 antibody. Anti-Rabbit IgG was used as a negative control. (left panel). Endogenous MCF-7 cellular proteins were also separated into cytoplasmic, chromatin, and soluble nuclear pools by fractionation methods and examined by western blot (right panel). Cleanliness of fractionation was determined by stripping membranes and re-probing with antibodies for TBP (nuclear) and SOD4 (cytoplasmic) proteins.
(TIF)

Figure S4 **Truncated FLAG-tagged PAD2 proteins reveal regions necessary for nuclear localization.** (A) The schematic at the top indicates the different truncated PAD2 proteins of 140, 278 and 437 amino acids. Truncated FLAG-PAD2 constructs were validated by overexpressing in HEK 293 cells with lysates analyzed by SDS-PAGE. Membranes were probed with an anti-FLAG antibody which detected predicted molecular weight of truncated proteins while β-actin shows loading control. (B) Truncation of PAD2 reveals regions of the protein necessary for subcellular localization by cellular fractionation. Truncated FLAG-PAD2 constructs were overexpressed in HEK 293 cells after which cellular proteins were separated by fractionation methods and examined by western blot. Cleanliness of fractionation was determined by stripping membranes and re-probing with antibodies for Histone H3 (nuclear) and SOD4 (cytoplasmic) proteins.
(TIF)

Figure S5 **PAD2 plays a role in gene expression in MCF-7 cells.** (A) PAD2 levels are significantly reduced in PAD2 knock down MCF-7 cells compared to shRNA controls. Whole cell lysates from shRNA control and PAD2 knock down MCF-7 cells were analyzed by western blot using an anti-PAD2 antibody and an anti-β-actin antibody for loading control.
(TIF)

Table S1 **Sequence of primers used for qPCR studies to validate microarray results and for ChIP assays.** Primers are listed in 5′–3′orientation.
(DOCX)
