# Supplementary figures and images for: Potential Role for PAD2 in Gene Regulation in Breast Cancer Cells
Source: PLoS One. 2012 Jul 24;7(7):e41242. doi: 10.1371/journal.pone.0041242 (PMC3404060; doi:10.1371/journal.pone.0041242)

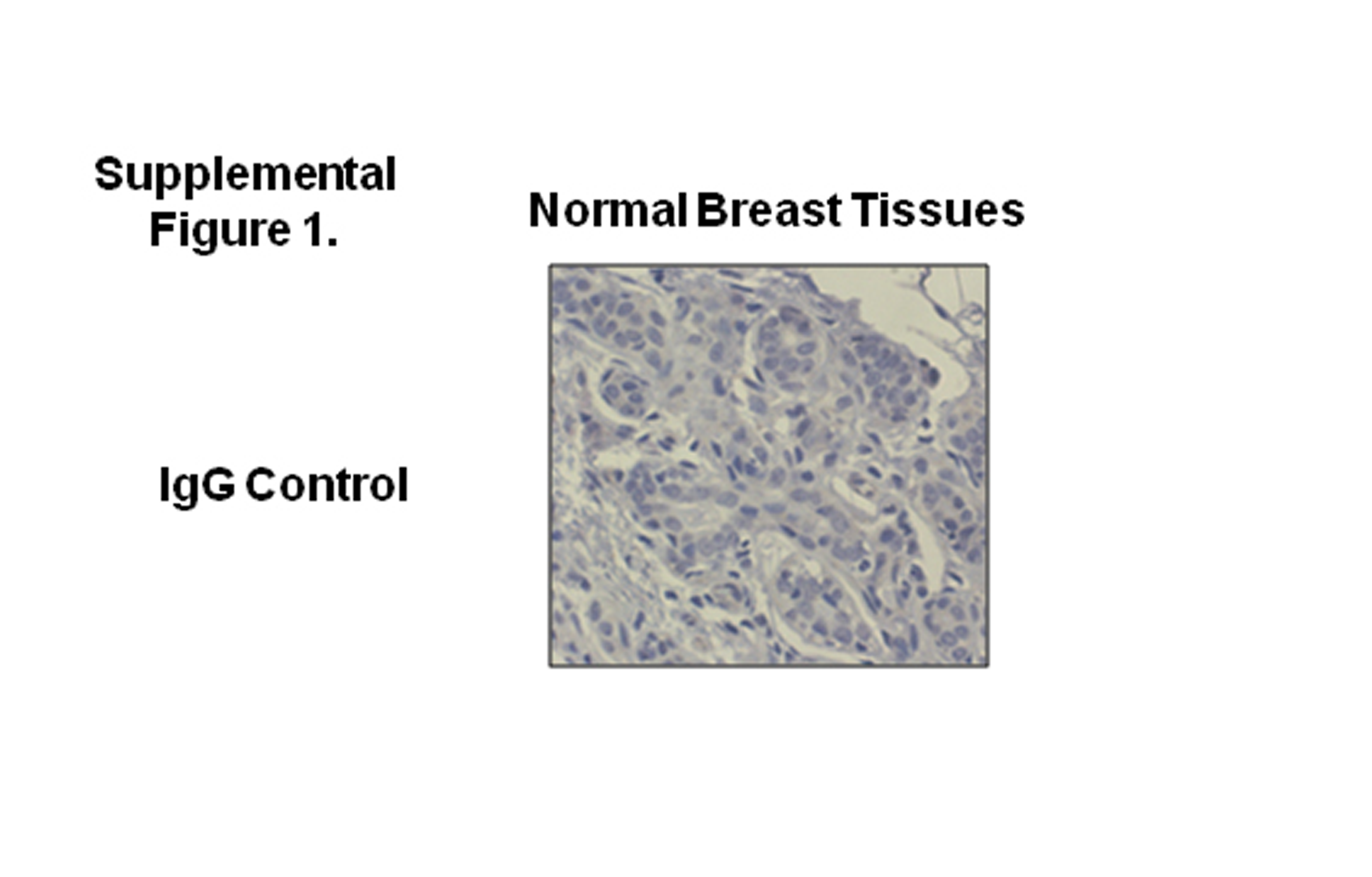

Supplement: Figure S1 — PAD2 staining in human mammary tissue is specific. As a control, human mammary tissue sections were probed with rabbit IgG at a concentration equal to that of primary PAD2 antibody and counterstained with hematoxylin. (TIF) [file pone.0041242.s001.tif]

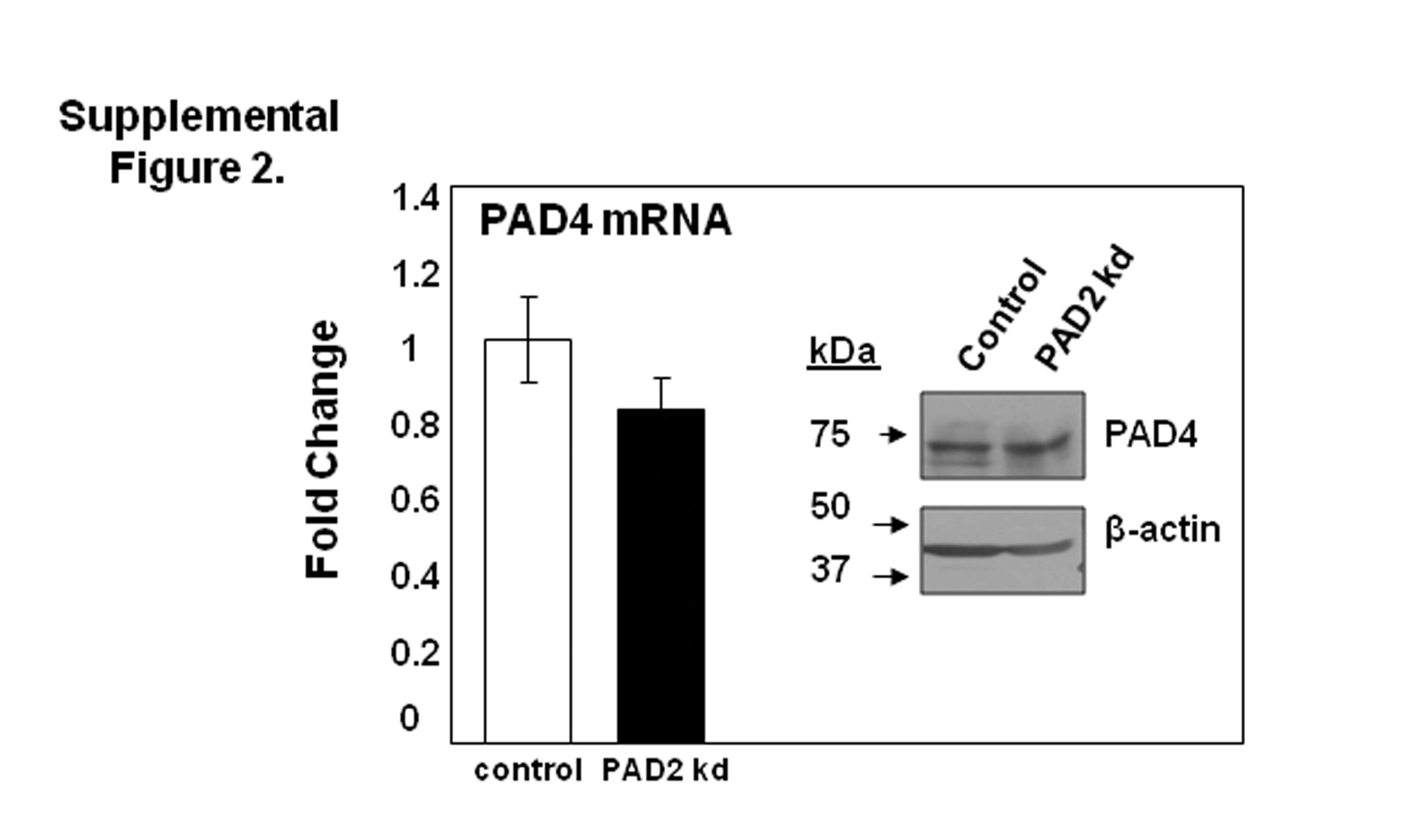

Supplement: Figure S2 — PAD4 expression levels are not altered in PAD2 knock down MCF-7 cells. Whole cell lysates from shRNA control and PAD2 knock down MCF-7 cells were analyzed by western blot using an anti-PAD4 antibody and an anti-β-actin antibody for loading control. RNA was purified from PAD2 knock down MCF-7 and shRNA control cells, reverse transcribed, and resulting cDNA used in qPCR reactions containing TaqMan assays to PAD4 and GAPDH as the reference gene control. Data represent the means ± SEM of four independent experiments performed in triplicate. All values are normalized to shRNA control samples and bars represent the means ± SEM. Means were separated by Student’s T-Test (P<0.01). (TIF) [file pone.0041242.s002.tif]

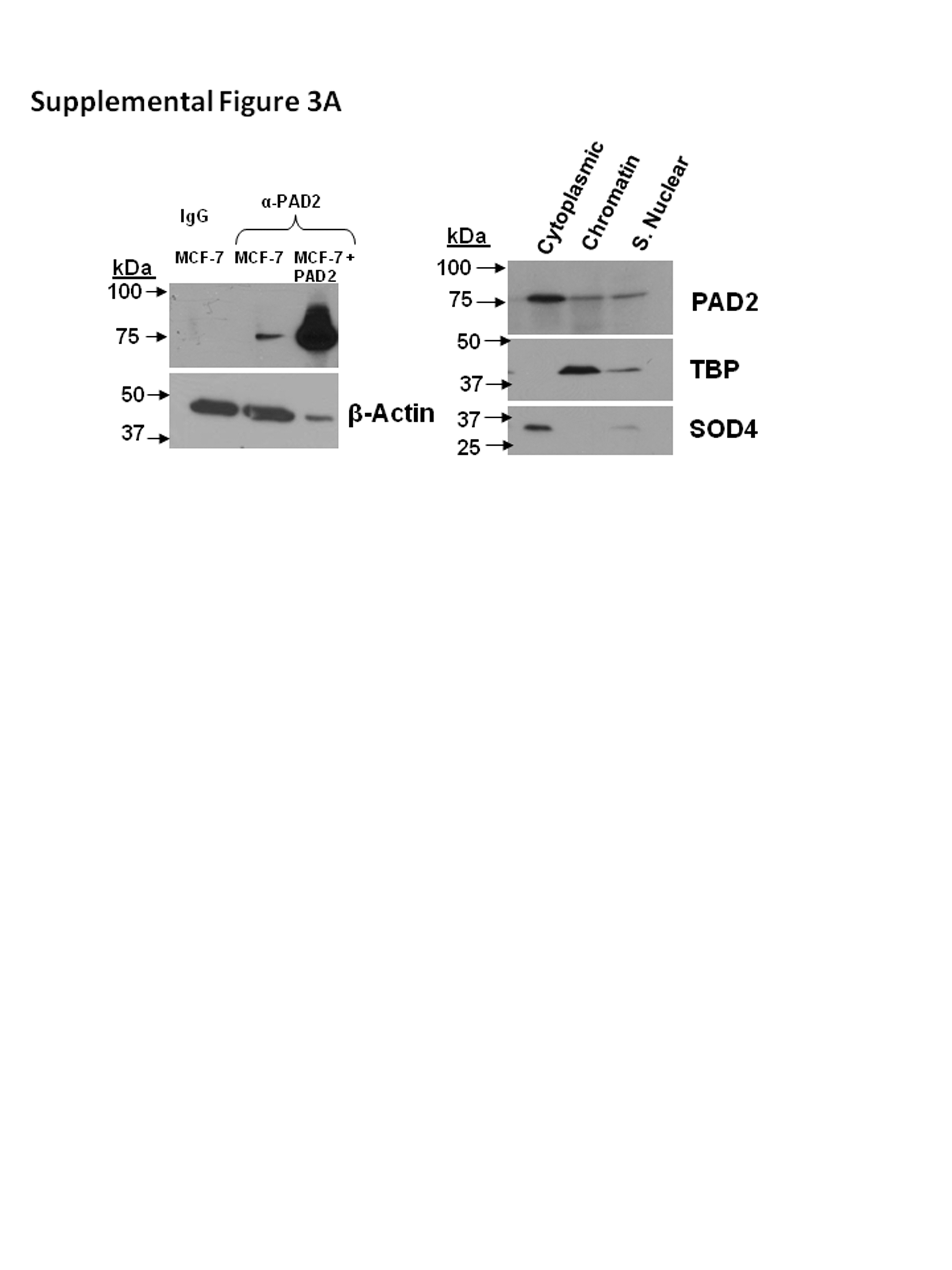

Supplement: Figure S3 — PAD2 associates with chromatin in the nucleus of MCF-7 cells. (A) PAD2 is endogenously expressed in MCF-7 cells and is detected in multiple cellular compartments. Wild type and PAD2 over-expressing MCF-7 whole cell lysates were subject to SDS-PAGE and probed with an anti-PAD2 antibody. Anti-Rabbit IgG was used as a negative control. (left panel). Endogenous MCF-7 cellular proteins were also separated into cytoplasmic, chromatin, and soluble nuclear pools by fractionation methods and examined by western blot (right panel). Cleanliness of fractionation was determined by stripping membranes and re-probing with antibodies for TBP (nuclear) and SOD4 (cytoplasmic) proteins. (TIF) [file pone.0041242.s003.tif]

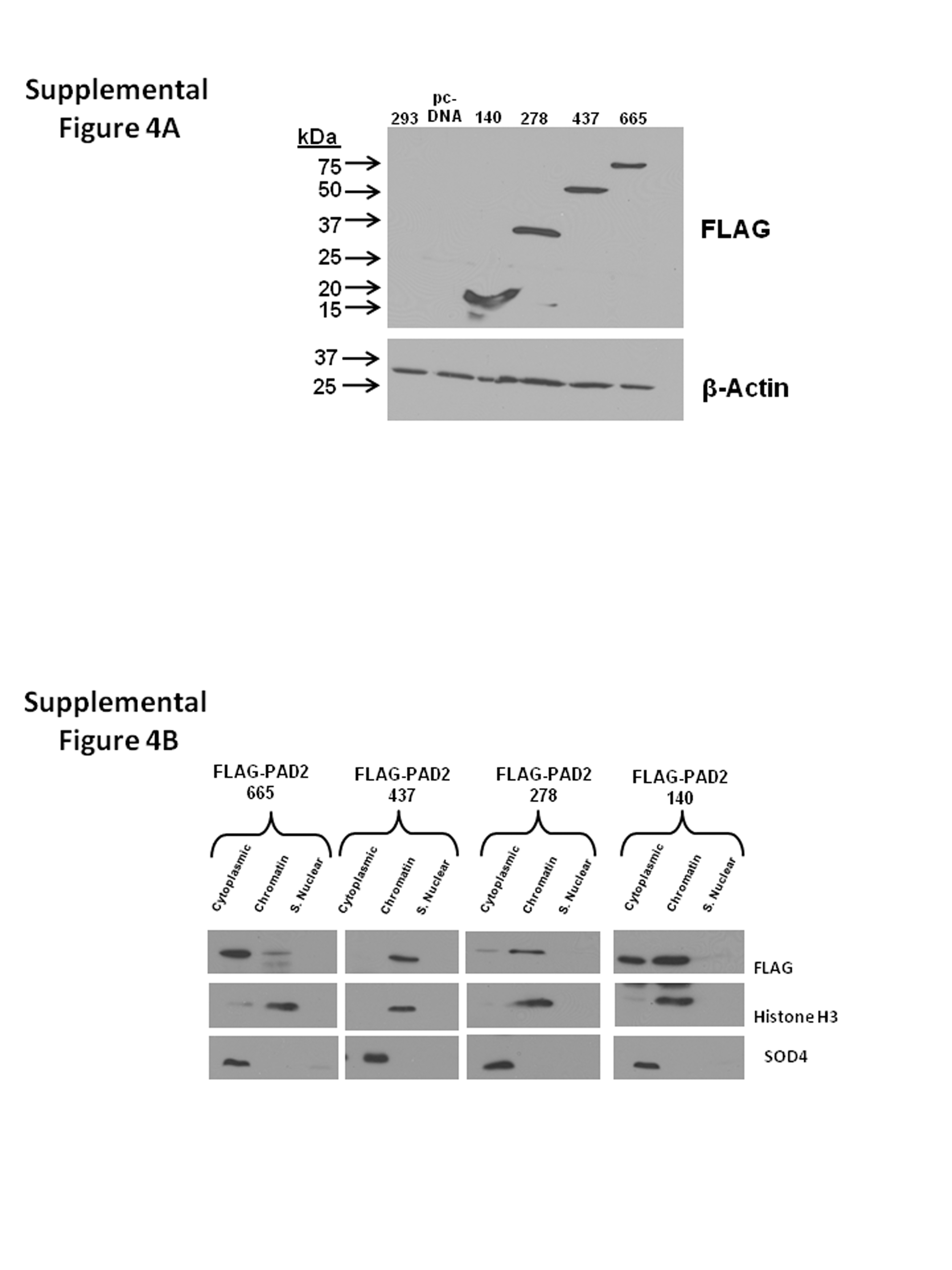

Supplement: Figure S4 — Truncated FLAG-tagged PAD2 proteins reveal regions necessary for nuclear localization. (A) The schematic at the top indicates the different truncated PAD2 proteins of 140, 278 and 437 amino acids. Truncated FLAG-PAD2 constructs were validated by overexpressing in HEK 293 cells with lysates analyzed by SDS-PAGE. Membranes were probed with an anti-FLAG antibody which detected predicted molecular weight of truncated proteins while β-actin shows loading control. (B) Truncation of PAD2 reveals regions of the protein necessary for subcellular localization by cellular fractionation. Truncated FLAG-PAD2 constructs were overexpressed in HEK 293 cells after which cellular proteins were separated by fractionation methods and examined by western blot. Cleanliness of fractionation was determined by stripping membranes and re-probing with antibodies for Histone H3 (nuclear) and SOD4 (cytoplasmic) proteins. (TIF) [file pone.0041242.s004.tif]

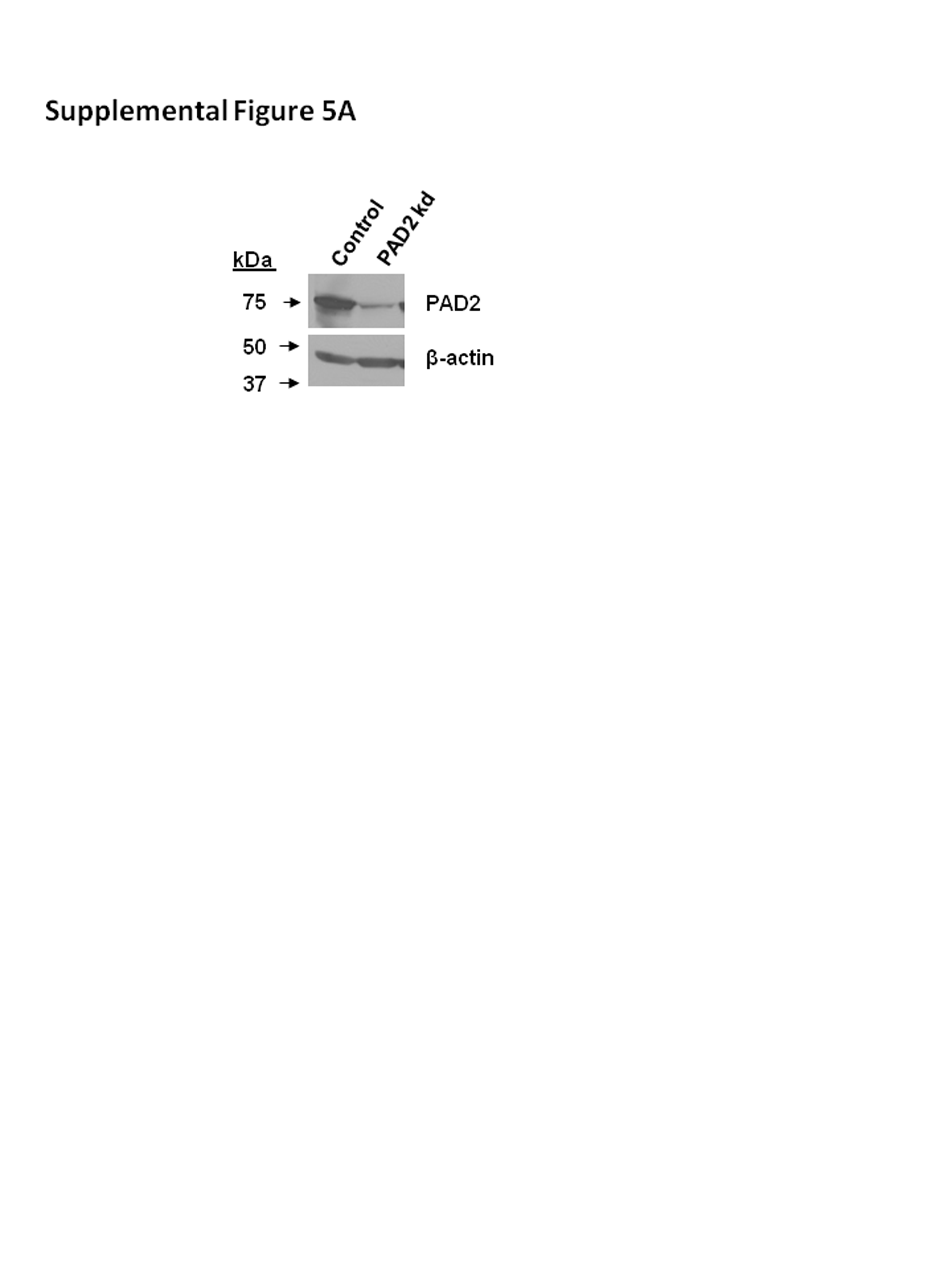

Supplement: Figure S5 — PAD2 plays a role in gene expression in MCF-7 cells. (A) PAD2 levels are significantly reduced in PAD2 knock down MCF-7 cells compared to shRNA controls. Whole cell lysates from shRNA control and PAD2 knock down MCF-7 cells were analyzed by western blot using an anti-PAD2 antibody and an anti-β-actin antibody for loading control. (TIF) [file pone.0041242.s005.tif]

Supplemental Table 1. Primers used for ChIP assays and qPCR validation of microarray studies.


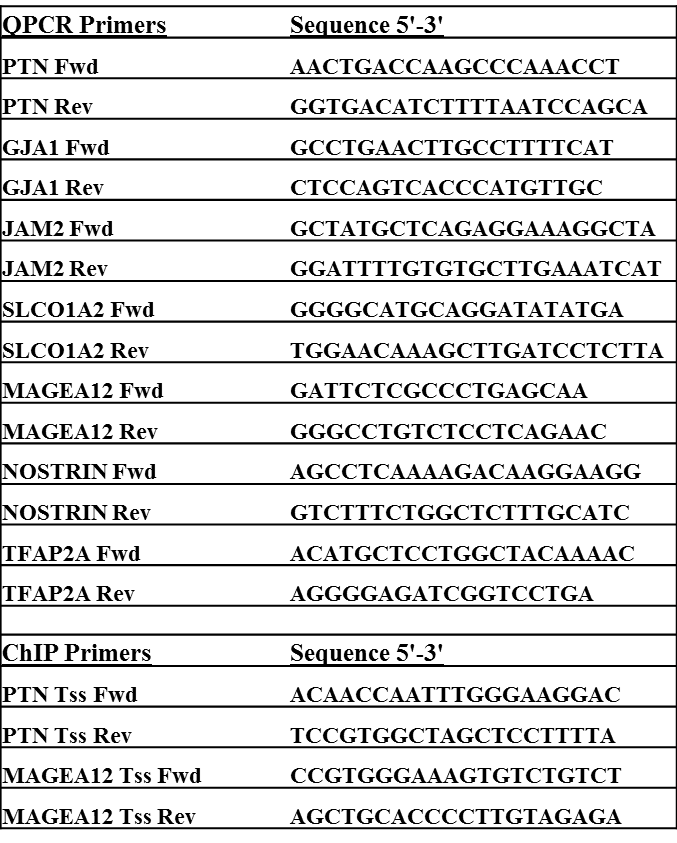

Supplement: Table S1 — Sequence of primers used for qPCR studies to validate microarray results and for ChIP assays. Primers are listed in 5′–3′orientation. (DOCX) [file pone.0041242.s006.docx]
